# Barriers to T Cell Functionality in the Glioblastoma Microenvironment

**DOI:** 10.3390/cancers16193273

**Published:** 2024-09-26

**Authors:** Noor E. Nader, Stephen C. Frederico, Tracy Miller, Sakibul Huq, Xiaoran Zhang, Gary Kohanbash, Constantinos G. Hadjipanayis

**Affiliations:** 1 School of Medicine, University of Pittsburgh, Pittsburgh, PA 15213, USA; nader.noor@medstudent.pitt.edu (N.E.N.); stephen.frederico@pitt.edu (S.C.F.); miller.tracy@medstudent.pitt.edu (T.M.); 2 Harvard Medical School, Boston, MA 02115, USA; 3 Department of Neurological Surgery, University of Pittsburgh, Pittsburgh, PA 15213, USA; huqs5@upmc.edu; 4 Sloan Kettering Memorial Cancer Center, New York, NY 10065, USA; zelbest@gmail.com

**Keywords:** glioblastoma, GBM, T cell, CNS, brain

## Abstract

**Simple Summary:**

Malignant brain tumors, such as glioblastoma (GBM), are devastating diagnoses for patients and their families, as these tumors are difficult to fully remove with surgery and respond poorly to chemotherapy and radiation. Immunotherapy is a novel approach to treating cancer, as these therapies are intended to initiate robust antitumor immune responses. However, immunotherapy for GBM has largely been unsuccessful. It is hypothesized that one of the major contributors to immunotherapy failure in GBM patients is the number of immunosuppressive blockades present in these patients. Often, the GBM microenvironment is characterized as highly immunosuppressive due to GBM recruiting anti-inflammatory immune cells to the microenvironment and releasing immunosuppressive factors such as PD-L1. Additionally, treatment given to GBM patients, such as corticosteroids, is immunosuppressive. In this review, we outline potential blockades to immunotherapy success in GBM patients to highlight where new approaches to combatting this malignancy should be considered.

**Abstract:**

Glioblastoma (GBM) is an aggressive primary brain tumor depicted by a cold tumor microenvironment, low immunogenicity, and limited effective therapeutic interventions. Its location in the brain, a highly immune-selective organ, acts as a barrier, limiting immune access and promoting GBM dissemination, despite therapeutic interventions. Currently, chemotherapy and radiation combined with surgical resection are the standard of care for GBM treatment. Although immune checkpoint blockade has revolutionized the treatment of solid tumors, its observed success in extracranial tumors has not translated into a significant survival benefit for GBM patients. To develop effective immunotherapies for GBM, it is vital to tailor treatments to overcome the numerous immunosuppressive barriers that inhibit T cell responses to these tumors. In this review, we address the unique physical and immunological barriers that make GBM challenging to treat. Additionally, we explore potential therapeutic mechanisms, studied in central nervous system (CNS) and non-CNS cancers, that may overcome these barriers. Furthermore, we examine current and promising immunotherapy clinical trials and immunotherapeutic interventions for GBM. By highlighting the array of challenges T cell-based therapies face in GBM, we hope this review can guide investigators as they develop future immunotherapies for this highly aggressive malignancy.

## 1. Introduction

Primary brain tumors are a leading cause of cancer-related death in adults, as these tumors are known to grow aggressively and often have a dismal prognosis. Glioblastoma (GBM) is considered the most aggressive of these tumors, as patients diagnosed with this disease have a median survival of just 15–18 months and a reduced quality of life [1]. GBM often grows in an infiltrative pattern throughout the brain, making this tumor difficult to fully resect with surgery, contributing significantly to its high recurrence rate. Additionally, treatment for this malignancy beyond surgical resection is quite limited, as GBM responds poorly to chemoradiation therapy. The creation of novel therapies targeted to GBM has been a challenge for the field, as GBM demonstrates a high degree of intertumoral heterogeneity, and the blood–brain barrier (BBB) blocks most therapeutic migration from the brain’s vasculature to the site of the tumor [2]. 

Immunotherapy has emerged as a promising treatment modality for non-central nervous system (non-CNS) cancers such as melanoma and leukemia; however, this approach has remained largely unsuccessful in the treatment of GBM [3,4]. Recently, three controlled phase III clinical trials assessing immune checkpoint blockade in GBM patients failed to lead to a survival benefit when compared to patients receiving bevacizumab or chemoradiation therapy [3,5]. Other forms of immunotherapy such as cancer vaccines and cellular therapies have been evaluated in GBM patients as well; however, most of these studies have not led to a survival benefit for patients. While these findings have largely been a disappointment for the field of neuro-oncology, several immunotherapy clinical trials in GBM have shown that subsets of patients do experience radiographic responses to treatment and a subsequent survival benefit [5]. These findings, as well as others, highlight the potential for immunotherapy in combatting GBM, and how the field must fully elucidate the blockades to therapeutic efficacy that exist in non-responding patients. 

Most immune-based therapies rely upon either host T cells, or those that are adoptively transferred into a patient, to carry out robust antitumor immune responses. Cytotoxic (CD8) T cells are initially primed with antigens in the lymphoid organs, which enable these cells to then engage in immune surveillance. Following priming, these cells can then become activated either in the lymphoid organs or peripheral tissues when antigenic epitopes released by a tumor bind the T cell receptor (TCR) and CD28 co-stimulation occurs, allowing for these cells to traffic to the site of the tumor and release granzymes and perforins. This ultimately leads to tumor cells undergoing apoptosis. 

Cancers, especially GBMs, utilize multiple mechanisms to inhibit the function of T cells, contributing to the failure of immunotherapy for these malignancies. Specifically, GBMs can recruit immunosuppressive cells, such as myeloid cells, to the tumor microenvironment (TME) that can release anti-inflammatory cytokines such as IL-10, leading to T cell exhaustion and immunosuppression [6]. Additionally, the GBM microenvironment is known to express high amounts of immune checkpoint ligands. These ligands are capable of binding to immune checkpoint receptors on the surface of T cells, reducing the ability of these cells to carry out antitumor immune responses. The combination of these factors, paired with hypoxia, create a GBM microenvironment that is immune-hostile and one that reduces therapeutic efficacy.

Beyond the immunosuppressive TME in GBM, treatment for this malignancy can also be immunosuppressive. Dexamethasone, a corticosteroid commonly given to GBM patients to control cerebral edema, has been shown to increase immune checkpoint expression, while chemoradiation has been reported in multiple studies to be immunosuppressive [7]. 

**Figure 1:**
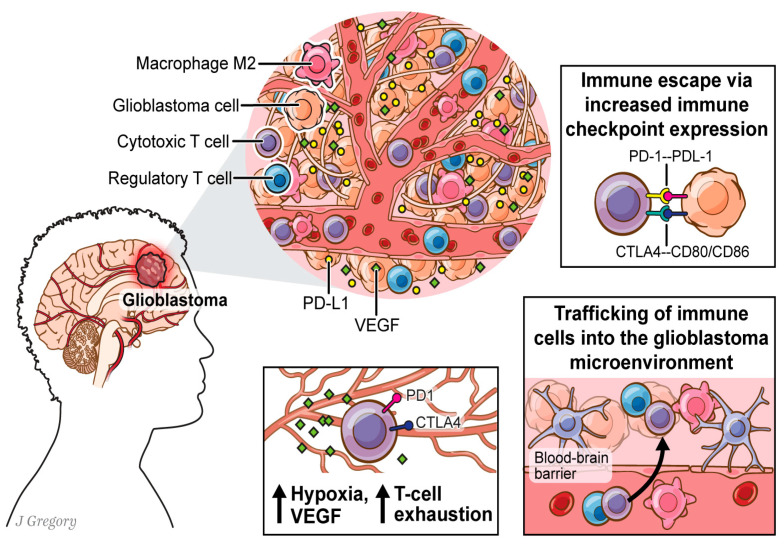
Drivers of immune suppression in the glioblastoma microenvironment.

In this review, we discuss the barriers to T cell-mediated anti-cancer immunity in GBM. Specifically, we discuss the T cell suppressing nature of current treatment modalities used for GBM, barriers to T cell trafficking, and the immunosuppressive nature of the GBM TME (Figure 1). We hope that by highlighting these barriers to T cell-mediated antitumor immunity, this review can outline the current challenges leading to immunotherapy failure in GBM patients, as well as highlight potential approaches the field may consider to surpass GBM treatment blockades in the TME.

## 2. Glioblastoma Treatment and Its Impact on T Cell Function

### 2.1. Corticosteroids 

Corticosteroids, such as dexamethasone, are reflexively prescribed to most patients with GBM due to their efficacy in reducing symptomatic cerebral edema. However, steroids are largely considered to have immunosuppressive effects due to their interaction with glucocorticoid response elements (GREs) leading to reduced transcription of pro-inflammatory cytokines, as well as these drugs’ ability to suppress nuclear factor kappa-light-chain-enhancer of activated B cells (NF-kB) [8,9,10]. Dexamethasone, a corticosteroid, is commonly prescribed to patients with GBM. Giles and colleagues explored the interaction between dexamethasone and checkpoint blockade therapy. Specifically, the team found that dexamethasone increased the amount of CTLA4 messenger ribonucleic acid (mRNA) and protein in CD4 and CD8 T cells and inhibited CD28-mediated cell cycle entry and differentiation [7,11]. This response largely affected naïve T cells, which served as a barrier to the differentiation of these cell into more specialized subset. Additionally, the team found that blocking CTLA4 or providing a strong CD28 co-stimulation prior to dexamethasone administration conferred resistance to dexamethasone, allowing for an increase in INFγ expression and an increase in survival in glioma-bearing mice treated with dexamethasone [7]. 

### 2.2. Surgery

While there has been a paucity of research on the impact of surgical resection on T cell-mediated antitumor immunity in cancer, there is evidence from non-CNS cancers that surgical trauma may promote an immunosuppressive stress response. Studies in non-CNS cancers have shown an increase in damage-associated molecular patterns (DAMPs) following surgical resection of a malignancy [12]. DAMPs can be nuclear or cytosolic proteins, extracellular matrix, or metabolic products [13]. It has been observed that the presence of some DAMPs enables an increase in IL-1β and IL-18, allowing for the recruitment of myeloid-derived suppressor cells (MDSCs) and macrophages to the TME [13,14]. 

In patients with breast cancer, fibroblasts can sense the presence of DAMPs, which leads to the activation of the NLRP3 inflammasome, enabling an upregulation of IL-1β secretion. It was found that this upregulation promoted tumor progression, which was partially combatted when NLRP3 or IL-1β was inhibited [15]. Multiple research groups have also observed that surgical trauma promotes an increased presence of T-regulatory cells (Tregs) as well as increased PD-L1 expression, which are both known for their highly immunosuppressive functions. In a study of patients with colon cancer, surgical trauma promoted colon cancer progression due to there being an increase in the amount of CCL18 expression following surgery [16]. Increased CCL18 expression can lead to immunosuppression, as this ligand can facilitate recruitment of Tregs to the TME and has been shown to polarize macrophages to an “M2” anti-inflammatory phenotype [16]. 

Further work is needed to understand how to combat the immunosuppressive effects of surgical resection. One study in lung cancer found that administration of anti-PD1 therapy following surgery reduced T cell apoptosis; however, similar mechanistic understanding is lacking and needed in GBM [17]. 

### 2.3. Chemotherapy 

Chemotherapy remains a central component of first-line therapy for GBM due to its demonstrated survival benefit, but chemotherapy is increasingly understood to have an immunosuppressive effect on the TME [18,19,20]. Temozolomide (TMZ), a DNA-methylating agent, is the current standard-of-care chemotherapeutic for GBM as part of the Stupp protocol. However, TMZ is associated with substantial lymphocyte toxicity with known side effects of lymphopenia and T cell dysfunction which are counterproductive for efforts seeking to engage the immune system [19,21,22,23]. Unsurprisingly, TMZ-induced lymphopenia and T cell dysfunction are associated with reduced overall survival in GBM [21,24,25]. 

In both CNS and non-CNS tumors, T cells exposed to chemotherapy display signs of mitochondrial damage characterized by an overall reduction in mitochondrial mass and volume. Furthermore, chemotherapy-exposed mature T cells have reduced response and function following ex vivo stimulation. Interestingly, TMZ cytotoxicity appears to be lymphocyte-specific, with no effect on monocyte counts [26]. In contrast to T cells, post-treatment monocytes retain their capacity to differentiate into mature dendritic cells [26]. Despite the lymphopenia induced by TMZ, Treg counts are not significantly impacted by chemotherapy’s cytotoxic effects and sustain their ability to suppress CD4 T cells [26]. Overall, this reduced T cell count and persistent Treg response strongly correlate with GBM progression and poor survival [21,25]. 

### 2.4. Radiation

Radiation therapy (RT) is another component of standard therapy for GBM that is known to confer a survival benefit but may simultaneously have detrimental effects on the immune system. Radiation slows tumor cell replication and induces cell death via apoptosis, necrosis, senescence, necroptosis, and ferroptosis [27,28]. However, radiation also promotes the development of tumor resistance and has been implicated in impairing T cell function. In fact, lymphocytes, including T cells, are among the most sensitive cells in the human body to radiation, with radiation known to cause 1.5- to -5-fold reductions in tumor-infiltrating T cells [29,30]. T cells that do survive radiation then upregulate the expression of exhaustion markers, including PD-1 and CD39, and exhibit diminished capacity for proliferation [29]. This hinders these cells’ differentiation into memory T cells [29]. Consequently, the exhausted T cells are unable to mount an effective antitumor response. It will be critical to develop therapies capable of restoring T cell effector function and promoting the recruitment of peripheral T cells necessary to form an immunogenic TME. However, achieving this in the context of GBM presents a specific challenge due to the inherent lymphopenia found in patients with GBM [27].

## 3. Barriers to T Cell Trafficking

### 3.1. Tumor Epigenetics

GBM immune evasion is driven in large part by epigenetic manipulation, a process that involves modulating gene expression by altering the accessibility of DNA sequences to the cellular transcription machinery, all without changing the underlying nucleotide sequence [31]. This is commonly accomplished via DNA methylation, histone modification, and chromatin remodeling [31]. Importantly, in addition to regulating tumor suppressor and cell cycle genes, epigenetic regulation also extends to cytokine and immune regulatory genes involved in orchestrating the immune response and immune cell trafficking. The nature of the elicited immune response depends on the cell type, activated signaling pathway, and cytokine repertoire involved. More specifically, cytokines allow for the initiation of immune responses, while chemokines mediate the recruitment and infiltration of immune cells to the specific site. A deficiency in these molecules inhibits the initiation of an immune response and the recruitment of immune cells.

Enhancer of Zeste Homolog 2 (EZH2) is a histone methyltransferase and a critical epigenetic regulator in many malignancies that primarily functions as an epigenetic silencer and is involved in the methylation of more than 200 tumor suppressor genes [32]. In GBM, EZH2 contributes to both tumor cell proliferation and migration by methylating tumor suppressor genes and disruption of the TME cytokine profile. Ratnam and colleagues found that blocking EZH2 activity in human GBM cells with GSK126, a global EZH2 methyltransferase inhibitor with good BBB penetrance, led to upregulation of CXCL9 and CXCL10 chemokines and subsequently elevated tumor infiltrating T cells [2,33]. Additionally, it was observed that treatment of immunosuppressed C57BL/6 GBM mice with GSK126 resulted in slower tumor growth [2]. Combination therapy with GSK126 and anti-PD1 therapy decreased growth 4-fold, enhanced the migration of T cells to the TME, and correlated with overall survival [2]. Combination therapy also increased CXCR3-expressing CD8 T cells in the draining lymph node [2]. In summary, EZH2 inhibition represents a promising strategy to restore pro-inflammatory cytokines and chemokines and potentially reverse T cell exclusion in GBM [2].

Beyond EZH2, there are other methylation patterns that have been associated with a reduction in the number of T cells present in GBM. Specifically, Dejaegher and colleagues compared immune infiltration between different GBM methylation patterns (RTK I, RTK II, IDH, and Mesenchymal) and found that the IDH methylation pattern was associated with the lowest levels of CD3 and CD8 T cell infiltration, while the mesenchymal methylation pattern had the highest overall levels of CD8 T cell infiltration [34,35]. In a separate methylation study, Tompa and colleagues found a high frequency of methylation for genes in the IL-7 signaling pathway—a critical pathway for cell survival and antitumor responses—with additional correlations found between IL-7 pathway hypermutation and GBM recurrence [36].

While tumor-specific epigenetic modifications can impact the ability of T cells to traffic to the tumor, epigenetic changes in T cells also critically influence cell state and antitumor activity. In fact, T cell exhaustion, driven by chronic antigen exposure, hypoxia, and T cell starvation, is predominantly mediated by epigenetic modifications [37]. In a murine model of liver cancer, CD8 T cells displayed unique chromatin remodeling (a form of epigenetic modification) specific to early exhaustion, or functional states, mainly within genomic regions of TCR signaling and cytokine production [38]. Moreover, it was observed that following prolonged exposure to the TME, tumor-infiltrating CD8 T cells with a functional state would gradually display similar chromatin patterns to dysfunctional T cells [38]. Taken together, these findings denote the critical implication of the epigenetic profiles of both tumor and immune cells in modulating the antitumor response and the potential for these modifications to be targeted therapeutically.

### 3.2. T Cell Sequestration

Another hallmark of GBM is T cell sequestration, referring to the phenomenon in which T cells are restricted to the bone marrow and are unable to migrate into the bloodstream and TME [27]. This may be mediated by Sphingosine-1-phosphate receptor 1 (S1P1), a G protein-coupled receptor expressed on endothelial and lymphoid cells that plays a critical role in the development of T cells in the thymus and in the modulation of lymphocyte trafficking. Both T cells and B cells expressing S1P1 egress from the secondary lymphoid organs to the periphery, whereas the absence of S1P1 expression is associated with bone marrow homing [39]. Loss of the S1P1 receptor on the T cell surface in GBM murine models was associated with bone marrow homing of T cells [39].

In humans, bone marrow-constrained T cells similarly have reduced expression of S1P1 compared to healthy controls [27]. Interestingly, following adoptive transfer, T cells engineered to express a stable form of S1P1 (S1P1-K1) did not home in the bone marrow but rather trafficked to the tumor site [27]. However, despite S1P1-K1 reversing T cell exclusion in the GBM tumor microenvironment and increasing CNS T cell count, significant overall survival was not observed. This was potentially attributed to the immunosuppressive nature of GBM, where immune checkpoint inhibition (ICI) combination therapy could potentially enhance T cell functions. In a murine model of GBM, those treated with a combination of S1P1-K1 and anti-PD-1 had 50% longer-term survival compared to mice treated with S1P1-K1 alone. Based on Chongsathidkiet et al.’s. discovery, the development of a mechanism restoring S1P1 surface expression is a potential therapy for promoting T cell response in GBM [27].

Furthermore, in the field of CAR-T cell therapy, both intravenous and intrathecal administrations of CAR-T cells have shown limited efficacy in GBM-specific clinical trials. To date, there have been few clinical trials on CAR-T cell therapy for GBM, none of which have advanced to phase III. CAR-T cell therapy involves modifying a patient’s own T cells to express a chimeric antigen receptor (CAR), which activates the cells upon binding to specific antigens on tumor cells, eliminating the need for secondary activation signals [40,41].

CAR-T cell therapy has demonstrated greater success in treating liquid tumors, benefiting from their accessible anatomy and high immunogenicity [42,43,44]. However, applying CAR-T cell therapy to GBM faces significant challenges, primarily due to the cold tumor microenvironment, the low immunogenicity of GBM tumors, and immune restriction within the brain [45]. As a result, this approach is likely to benefit only a small subset of GBM patients whose tumors express specific antigens.

Currently, there are several CAR-T trials targeting GBM tumors (see Table 1) expressing EGFRvIII, IL13Rα2, HER2, B7-H3, Chlorotoxin, CD133, EphA2, IL7R1, NKG2D, CD70, IL8, and GD2 in newly diagnosed, advanced-stage, and recurrent tumors [45,46,47,48,49,50,51]. A recent CAR-T trial for GBM (NCT02209376) was terminated after 8 months, as researchers observed increased expression of regulatory receptors and infiltration of regulatory T cells in the tumor following CAR-T cell infusion [45]. Additionally, they noted a decrease in EGFR receptor levels in five out of seven patients. Despite these outcomes, the trial demonstrated the feasibility of CAR-T cell trafficking into GBM tumors [45].

In a phase I pilot study (NCT05168423) evaluating CAR-T-EGFR-IL13Ra2 cells, pseudo-tumor progression was observed a month post-infusion; however, overall tumor size decreased by two months post-infusion [49]. Initial results suggest a promising effect of CAR-T-EGFR-IL13Ra2 cell therapy in GBM and combining it with immune checkpoint blockade (ICB) may lead to significant tumor regression and improved overall survival [49].

Similarly, in another phase I study (NCT03170141) of GD2-specific 4SCAR-T cells in GBM patients, pseudo-tumor progression was observed following CAR-T cell infusion, followed by tumor regression in some patients [48]. Despite an increase in tumor immune infiltration, antigen loss in tumors was also noted. However, the clinical benefit assessment was limited due to the study’s small sample size [48].

Overall, clinical trials of CAR-T cell therapy in GBM have shown promising early responses that diminish over time due to the immune-suppressive tumor microenvironment and tumor evasion mechanisms. These clinical studies collectively highlight the feasibility of CAR-T cell trafficking into GBM tumors yet emphasize the need for new strategies to overcome tumor antigen loss post-infusion.

### 3.3. Blood–Brain Barrier

The brain is uniquely protected by a specialized network of microvasculature referred to as the blood–brain barrier (BBB). This network of specialized vessels features endothelial cells sealed by tight junctions, granting selective permeability. Functionally, the BBB serves as a physiological defense barrier of the brain that tightly regulates the traffic of substances and cells to and from the CNS. Under normal conditions, the BBB prevents the entry of immune cells due to lack of expression of cell adhesion molecules necessary for immune cell trafficking [52]. However, under certain conditions of inflammation, the BBB selectivity against immune cells is disrupted through the expression of cell adhesion molecules (CAMs) or loss of tight junctions. This disruption allows peripheral immune cells, such as activated T cells, to infiltrate the brain parenchyma. Inflammatory cytokines are key mediators in facilitating the migration of immune cells into the CNS by inducing the expression of CAMs on BBB endothelial cells [53]. However, in GBM, the immunosuppressive TME downregulates the expression of pro-inflammatory CAMs, presenting a challenge for drug delivery and immune cell extravasation.

Another recently described relevant chemokine is LIGHT (TNFSF14), which plays a critical role in vasculature formation, T cell priming, and infiltration in the TME and secondary lymphoid organs [54,55]. Bienkowska and colleagues demonstrated that treatment of an orthotopic GBM mouse model with an adeno-associated viral vector (LIGHT-AAV) enhanced cytotoxic and memory T cell infiltration and improved the overall antitumor response [56]. Mice treated with immune checkpoint blockade in addition to LIGHT-AAV experienced enhanced survival [56]. Interestingly, blocking the LIGHT receptor LTBR not only reduced the number of tertiary lymphoid structures, but also reduced the number of stem-like CD8 T cells [56]. Taken together, these findings suggest that strategies aimed at local T cell infiltration and priming may offer a promising treatment avenue for GBM.

## 4. The Immunosuppressive Tumor Microenvironment

### 4.1. MDSCs

Myeloid-derived suppressor cells (MDSCs) in the GBM microenvironment are a specialized population of immune cells that play a crucial role in modulating the immune response within the tumor. MDSCs are myeloid lineage-derived and are typically characterized by their ability to suppress the activity of various immune cells, particularly T cells and natural killer cells. Specifically, it has been well established that the availability of arginine is correlated with increased amounts of T cell proliferation, and that MDSCs suppress T cell function by expressing high levels of the enzyme arginase [57]. Arginase activity leads to increased catabolism of arginine, subsequently depleting it in the microenvironment and enabling a downstream inhibition of T cell proliferation [58,59].

Studies in non-CNS malignancies have demonstrated a pivotal role for MDSCs in creating an immunosuppressive TME with subsequent correlations with poor patient outcomes [60,61]. These MDSCs are recruited to the TME in response to various factors, including tumor-derived signals, such as cytokines and chemokines, including granulocyte-macrophage colony-stimulating factor (GM-CSF) [62]. Drivers of MDSC effects in the TME can be categorized as related to MDSC expansion or MDSC activation. Factors driving MDSC expansion include COX2, prostaglandins, SCF, M-CSF, IL-6, GM-CSF, and VEGF, converging on the JAK-STAT3 pathway as a critical regulatory mechanism. STAT3 is a key transcription factor facilitating MDSC survival and proliferation while inhibiting their differentiation into mature myeloid cells [63]. S100A8 and S100A9 proteins, induced by STAT3, contribute to MDSC expansion and migration to the tumor site.

Factors that induce MDSC activation include inflammatory mediators such as IFNγ, Toll-like receptor ligands, IL-13, IL-4, and TGFβ, engaging various signaling pathways involving STAT6, STAT1, and NF-κB. The complex interplay between MDSCs and their microenvironment varies depending on the disease context, emphasizing the multifaceted nature of MDSC-mediated immune suppression [57].

There is a growing body of evidence indicating that MDSCs play a direct role in supporting tumor development, neovascularization, and metastasis. For example, MDSCs have been shown to produce factors such as VEGF and basic fibroblast growth factor (bFGF) that promote tumor neo-angiogenesis [64]. MDSCs also play a crucial role in tumor-mediated immunosuppression through several mechanisms, including depriving T cells of essential amino acids necessary for proliferation and antitumor reactivity, as well as producing nitric oxide (NO) and ROS that damage T cell receptors and induce apoptosis. These cells also secrete immunosuppressive cytokines like interleukin-10 (IL-10) and transforming growth factor-beta (TGF-β1), and upregulate programmed death-ligand 1 (PD-L1) to inhibit T cell reactivity. Additionally, MDSCs can reduce T cell receptor ζ-chain expression and produce growth factors and cytokines that stimulate tumor growth and suppress immune responses [65].

In recent years, significant progress has been made in therapeutically targeting MDSCs in various cancers, with strategies focusing on inhibiting MDSC immunosuppressive activity, blocking their recruitment to the tumor site, and modulating myelopoiesis or depleting MDSCs within tumor-bearing individuals. For instance, inhibitors like sildenafil and tadalafil have shown efficacy in reducing MDSC functions by downregulating NOS2 and ARG1 activities, leading to an enhancement in antitumor immunity [66]. The histone deacetylase inhibitor entinostat, in combination with anti-PD-1 antibodies, has been shown to improve survival and reduce tumor growth in murine models of lung and renal cell carcinoma [67]. Finally, various methods are being utilized to deplete MDSCs, such as using all-trans retinoic acid (ATRA), which differentiates MDSCs into mature myeloid cells, or low-dose chemotherapy, which selectively induces MDSC apoptosis [68,69].

In GBM specifically, Lathia and colleagues observed that treatment with Ibudilast, which is a brain-penetrant MIF-CD74 interaction inhibitor (an axis that MDSCs rely heavily upon), reduced the functionality of MDSCs and enhanced the activity of CD8 T cells in the GBM microenvironment [70]. These approaches may hold promise in improving cancer immunotherapy by mitigating MDSC-mediated immunosuppression [68,70].

### 4.2. M1/M2 Macrophages

Macrophages are generally accepted to be polarized into one of two forms: M1 or M2, each with opposing activities. M1 macrophages contribute to T cell proliferation and promote tissue damage primarily through the production of nitric oxide (NO), mediated by iNOS. These cells are typically considered to be “pro-inflammatory”. In contrast, M2 macrophages support tissue repair and cell growth, with ornithine production catalyzed by arginase as a key factor, causing these cells to be considered more “anti-inflammatory”. M1 and M2 macrophages are closely associated with Th1 and Th2 immune responses, and their functions can be influenced by immune response products like IFN-γ and IL-4. This delicate balance between M1 and M2 macrophages underlines the intricate connection between innate and adaptive immunity [71].

Recent research challenges the conventional belief that adult resident tissue macrophages solely originate from the bone marrow. In fact, most tissue macrophages come from yolk sac progenitors. This distinction has been observed in glioma mouse models, where resident yolk sac-derived microglia and recruited bone marrow-derived tumor-associated macrophages (TAMs) behave differently and respond differently to anti-macrophage therapies targeting CSF1 signaling [72]. TAMs in brain cancers exhibit significant heterogeneity. Single-cell analyses have revealed that TAM compositions differ in primary brain tumors (e.g., GBM) compared to metastatic brain tumors, with microglia prevalent in newly diagnosed GBM and macrophages in recurrent GBM. TAM heterogeneity is influenced by genetic alterations in glioma cells, such as mutations in genes like NF1, PTEN, and IDH1. Epigenetic changes in glioma cells also affect TAM infiltration, with treatments like radiation therapy altering TAM composition. Additionally, TAM heterogeneity shows sex-specific differences, with male GBM mouse models and patients displaying distinct characteristics in microglia [73].

MDSCs have been identified as a significant precursor of TAMs, contributing to the overall immunosuppression within the TME. Recent findings underscore the critical role of recruiting circulating inflammatory monocytes through various factors such as chemokines like CCL2 and CCL5, cytokines like CSF-1, and members of the VEGF family. In this context, complement components, especially C5a, play a pivotal role in the recruitment and functional polarization of TAMs, influencing their ultimate phenotype. Furthermore, the interplay of CSF-1 acts as a key factor in attracting monocytes, leading to the survival and polarization of TAMs toward the immunosuppressive M2 macrophage phenotype. Conversely, granulocyte-macrophage colony-stimulating factor (GM-CSF) serves to activate macrophages with antitumor functions. This complex network of recruitment and polarization mechanisms contributes to the diverse roles that TAMs play within the TME [74].

Recently, single-cell technologies have revealed novel functional states of TAMs in GBM. These states include immunosuppressive macrophages expressing genes such as Ccl22, Cd274 (encoding PD-L1), and Ccl5. Macrophages and microglia also exhibit distinct functional states in GBM, with macrophages showing immunosuppressive features and activated metabolic pathways. Furthermore, single-cell analysis has identified multiple molecular subtypes of myeloid cells in GBM, highlighting the diversity of TAM functional states. These findings offer the potential for personalized therapeutic strategies targeting specific TAM states in GBM patients [74].

Given the significant roles of TAMs within the GBM TME, researchers are exploring the potential of targeting TAMs as a strategy for GBM treatment. This approach primarily focuses on preventing the recruitment of TAMs to the tumor site. Several clinical trials are currently underway, investigating inhibitors that target key proteins associated with TAM recruitment. One such target is the colony-stimulating factor 1 receptor (CSF1R), and inhibitors like pexidartinib have shown promise. Another target is angiopoietin-2 (ANG2), a protein involved in vascular destabilization in the hypoxic TME. Clinical trials using ANG2 inhibitors, such as MEDI3617 and trebananib, have reported limited success and notable adverse effects. A third target is CXCR4, a receptor involved in tumor growth and TAM recruitment. Plerixafor, a CXCR4 inhibitor used in combination with radiation therapy, has shown promise in extending overall survival. However, clinical trials are ongoing to optimize this approach [75].

### 4.3. Tregs

Regulatory T cells (Treg cells) are a subset of immune cells that prevent the immune system from attacking the body’s own tissues, maintain self-tolerance, and suppress excessive immune responses. Treg cells are identified by CD25 and the transcription factor Foxp3. They can also hinder antitumor immune responses in cancer. Treg cells frequently infiltrate the TME in both human and murine cancers, fostering an immune-suppressive TME characterized by the presence of MDSCs, TAMs, and immune checkpoint molecules. The migration of Treg cells from the thymus to the TME is orchestrated by chemokine gradients, with receptors like CCR4, CCR8, CCR10, and CXCR3 responding to different chemokines. Treg cells have a tendency for recognizing self-antigens and expand within the TME due to the abundance of tumor-associated self-antigens. The proliferation of Treg cells is further fueled by immunosuppressive cytokines like TGF-β and IL-10, produced by both tumor cells and immune cells within the TME [76].

Treg cells employ several mechanisms to suppress immune functions within the TME. These mechanisms include CTLA4-mediated inhibition of APC function, limiting T cell activation by consuming interleukin (IL)-2, producing inhibitory cytokines such as TGF-β, IL-10, and IL-35 to hinder effector T cell activation, releasing cytotoxic substances (perforin and granzyme) to induce effector T cell apoptosis, and expressing immune checkpoint molecules like CTLA4, ICOS, and LAG3, which inhibit the cytotoxic functions and proliferation of effector T cells. Additionally, the PD-1 pathway, expressed by both activated effector Treg cells and effector T cells, plays a role in immune regulation. Lastly, the generation of immune-suppressive metabolites, including indoleamine 2,3-dioxygenase and adenosine, further contributes to immune suppression and T cell dysfunction in the TME. These collective mechanisms underscore the significant impact of Treg cells on modulating immune responses in the TME [76].

The impact of tumor-infiltrating Treg cells on clinical outcomes in various cancer types is still a subject of debate. While studies have shown that high Treg cell infiltration is associated with poor survival in some cancers such as ovarian, breast, and hepatocellular carcinomas, there are conflicting findings in other cancer types, including head and neck cancers and Hodgkin’s lymphoma, where Treg cell density has shown an inverse correlation with immune control and overall survival. These discrepancies may result from imperfect markers to identify suppressive cells, technical differences, or unique tumor microenvironments [77]. The antigen specificity of Treg cells remains unclear, making their effects context-dependent, potentially acting as inhibitors of antitumor immune responses or as controllers of chronic inflammation in different tumor settings [77]. Nevertheless, CD4+ T cell populations, including Tregs, are commonly found in glioma specimens and increase as the tumor grade progresses. Foxp3+ Tregs are rare in low-grade oligodendroglioma-type tumors but more common in high-grade astrocytic gliomas. This variation suggests a link between tumor grade and Treg presence in gliomas [78].

Research has demonstrated that the removal of Tregs is advantageous for treating GBM, emphasizing the significance of targeting and eliminating Tregs within the GBM microenvironment as a pivotal aspect of GBM treatment. A previous study revealed that the removal of Tregs from GBM patients results in the restoration of normal T cell proliferation and cytokine responses. In vitro depletion of Tregs from peripheral blood leads to the reinstatement of effector T cell function, increased T cell proliferation, and a shift from a Th2 to a Th1 cytokine profile, underscoring the significant role of Tregs in glioma-induced immune suppression [79].

Strategies to eliminate Tregs often aim to reduce Treg induction and peripheral recruitment, thereby alleviating Treg-mediated inhibition of effector T cells and boosting their antitumor activity. These approaches involve focusing on high-affinity IL-2 receptors and CD25, which are expressed on Tregs. The administration of anti-CD25 and the utilization of personalized platforms, such as the platelet-rich fibrin patch (PRF-P), can aid in the removal of Tregs. This, in turn, diminishes their suppressive impact on effector T cells and enhances the body’s ability to mount an antitumor response [80].

In vivo labeling of glucocorticoid-induced tumor necrosis factor-related protein (GITR) in naive or tumor-bearing mice has revealed that Treg cells constitutively express higher levels of GITR than conventional T cells. Therefore, GITR serves as an immune checkpoint expressed in Treg cells, and its activation by its ligand enhances proliferation and effector function in CD4 effector cells and cytotoxic T lymphocytes while destabilizing and depleting Treg cells and reducing their suppressive function [81]. In a 2021 study led by Amoozgar and colleagues, anti-GITR treatment in murine GBM models demonstrated its ability to transform the immunosuppressive GBM TME [82]. This transformation primarily involved the targeting of GBM Treg cells, which were converted into antitumor Th1-like CD4 T cells. Notably, this approach offered the advantage of tumor-specific treatment potential without inducing systemic autoimmunity. The study extensively explored the roles of Treg cells in the GBM immune TME, their response to immune checkpoint inhibitors, and strategies for overcoming resistance to anti-PD1 therapy. Importantly, the anti-GITR treatment selectively homed in on GBM Treg cells, heightening the tumor’s responsiveness to anti-PD1 therapy. Altogether, this study provides valuable insights into surmounting GBM immunotherapy resistance and highlights the promise of potential combination therapies involving anti-PD1/PDL1 and the standard of care for GBM patients characterized by high Treg cell accumulation.

### 4.4. Hypoxia

Hypoxia is a hallmark of many cancers and is prevalent in the GBM microenvironment, particularly within the tumor core. It is primarily driven by poor neovascularization and high oxygen consumption by tumor cells. As a significant driver of immunosuppression, drug resistance, and poor survival in GBM, hypoxia prompts GBM tumors to alter their metabolic programs and rely on autophagy to recycle and eliminate damaged cellular components [83,84,85]. Additionally, immune cells are also highly sensitive to the hypoxic environment within GBM tumors, with T cells shifting to an exhausted state under such hypoxic stress [86].

Bevacizumab, an anti-vascular endothelial growth factor (VEGF) therapy, has been explored for treating GBM due to the role of hypervascularity in promoting GBM progression and immune evasion through hypoxia [87,88,89]. The rationale behind using anti-VEGF therapy like bevacizumab is to normalize tumor vasculature, potentially improving drug delivery and enhancing treatment efficacy.

However, bevacizumab’s effectiveness in GBM treatment remains controversial despite being the only FDA-approved anti-VEGF drug for this indication [90,91,92,93]. While some clinical trials have demonstrated benefits such as radiographic response and improved progression-free survival, these benefits rarely translate into overall survival benefit [93,94,95]. Moreover, bevacizumab is associated with significant side effects and toxicities, which have been a concern in clinical practice and trials.

In many clinical trials, bevacizumab has not progressed beyond phase II testing (68 trials) for various reasons, including concerns over toxicity and inconsistent clinical outcomes [96,97,98]. All trials involving bevacizumab alone or in combination with chemotherapy, radiation, or immunotherapy, have shown no survival benefit. Only five trials have advanced to phase III with results available (NCT03149003, NCT02017717, NCT00943826, NCT05718466, and NCT00884741) [3,94,95,99,100]. A phase II trial (NCT02337491) assessing the therapeutic benefit of pembrolizumab with and without bevacizumab revealed no survival benefit associated with either arm [51]. However, worsened survival was associated with dexamethasone use, plasma VEGF levels, mutant IDH, and unmethylated MGMT. Additionally, a phase III trial (NCT00943826) evaluating TMZ and radiation with and without bevacizumab in newly diagnosed GBM patients demonstrated no survival benefit from adding bevacizumab to the treatment regimen [94].

Despite its theoretical potential to normalize vasculature and improve drug delivery, the practical limitations of BBB penetration and the complexities of GBM biology have hindered its success as a standard therapy. The failure of bevacizumab further emphasizes the importance of developing GBM-specific therapeutic targets rather than relying on drugs that have been successful in other tumor types.

Interestingly, hypoxia is also linked to greater PD-L1 expression and appears to impact Tregs and effector T cells quite differently. Specifically, while hypoxia induces terminal exhaustion in CD8 effector T cells, it has also been shown to promote immunosuppressive Treg activity and homing in the TME [84,101]. This immunosuppressive activity is associated with CD39, which is expressed as part of the HIF pathway and is involved in immune regulation [84,101].

Furthermore, hypoxia contributes to mitochondrial dysfunction, specifically through Blimp-1 activation and the accumulation of reactive oxygen species (ROS), which subsequently limits the T cell antitumor response [86]. In vitro experiments have demonstrated that, under hypoxic conditions, T cells undergo cellular reprogramming involving the activation of the HIF pathway, which is crucial for maintaining cellular oxygen homeostasis [86]. However, regardless of HIF expression, CD8 T cells experience mitochondrial mass loss under hypoxic conditions, which has been correlated with Blimp-1 activation [102]. Notably, blocking Blimp-1 activity in CD8 T cells has been shown to restore their antitumor function under hypoxic conditions [102]. Overall, there is a complex interplay between hypoxia, T cell function, and the antitumor T cell response. Future therapeutic strategies should focus on targets such as Blimp-1 and CD39 to potentially restore a robust T cell response in GBM.

## 5. Immune Checkpoints

### 5.1. PD1/PDL1

Programmed cell death protein 1 (PD-1) and PD-L1 are critical proteins that are part of the immune checkpoint pathway, which in normal states helps prevent the immune system from attacking the body’s healthy cells. PD-1 is a receptor protein found on the surface of certain immune cells, including T cells, whereas PD-L1 is a ligand protein expressed on the surface of various cells, including cancer cells. When PD-1 on immune cells binds to PD-L1, it sends inhibitory signals that suppress the immune response. Cancer cells hijack this interaction between PD-1 and PD-L1 to evade the immune system. Inhibition of this checkpoint inhibitor pathway is an area of significant interest and promise for immunotherapy. Wei and colleagues found that the strength of PD-1 signaling had differential effects on T cell functions with strong PD-1 signaling-impairing T cell effector functions, including reduced proliferation, cytokine production, and cytotoxicity. In contrast, weak PD-1 signaling had a milder impact on T cell activities [103]. This suggests that PD-1 acts as a rheostat, where the strength of the signal it provides modulates the degree to which T cells are inhibited [103]. Xue and colleagues emphasized the intricate nature of PD-L1 expression in gliomas, its potential involvement in immune evasion, and its variable prognostic significance. They underscored the necessity for standardized assessment methods and further research to elucidate its role in the context of glioma. The study, which examined PD-L1 at both the mRNA and protein levels, detected PD-L1 expression in glioma cell lines and tumor tissues. It revealed substantial variability in PD-L1 expression across different studies, with positive rates in glioma samples showing considerable diversity. This diversity is attributed to differences in sample sizes, pathological grades, tissue preparation techniques, and diagnostic criteria. Moreover, the research identified a link between PD-L1 expression and glioma grade, indicating its potential as a tissue biomarker for gliomas. High-grade gliomas exhibited more pronounced and widespread PD-L1 staining, implying that PD-L1 may play a role in immune evasion and the progression of malignant gliomas [104].

A recent meta-analysis involving 9 studies with 806 GBM patients demonstrated a significant association between PD-L1 expression in tumor tissues and reduced overall survival, as well as a trend linking PD-L1 expression to the IDH1 gene mutation status. This suggests the prognostic and therapeutic potential of this checkpoint inhibition pathway in GBM [105]. These findings, as well as others, have led to the initiation of many immune checkpoint inhibitor clinical trials in GBM (see Table 2). Notably, while many ongoing clinical trials in GBM are focused on PD1/PD-L1 blockade, other immune checkpoints are now being targeted as well.

PD-1/PD-L1 inhibitors are a category of immunotherapy medications engineered to disrupt the interaction between PD-1 and its corresponding ligand, essentially unleashing the immune system by removing its regulatory brakes. By inhibiting PD-1 or PD-L1, these drugs can enhance the body’s immune response against cancer cells, which has shown promising results in various types of cancer treatment, such as melanoma, lung cancer, and more [106]. Researchers are also exploring their potential in GBM and other challenging cancers. However, clinical studies of anti-PD-1/PD-L1 monotherapy in GBM have generally shown limited effectiveness. Pembrolizumab, an FDA-approved drug for various cancers, including melanoma, did not significantly improve GBM patient survival when used alone. Similar results were observed in studies involving high-grade gliomas (WHO grade 3 and 4).

Nivolumab, another widely used checkpoint inhibitor, also failed to significantly extend survival in GBM patients when compared to a bevacizumab-treated control group. Atezolizumab and durvalumab, both anti-PD-L1 antibodies, showed limited clinical benefits in GBM patients, with some exceptions, but the overall efficacy of checkpoint inhibitor monotherapy for GBM remains unsatisfactory [107]. Neoadjuvant therapy with PD-1/PD-L1 checkpoint inhibitors has demonstrated promise, enhancing overall survival, improving immune responses, and modifying gene expression profiles [108]. Combination strategies involving checkpoint inhibitors, temozolomide, radiation therapy, and other immunotherapies show potential, although ongoing research seeks to optimize these approaches. Prioritizing safety, monitoring immune-related adverse events, and managing glucocorticoid use are essential. These combined efforts offer hope for improved GBM therapy [107].

### 5.2. CTLA4

Another key immune checkpoint protein is cytotoxic T-lymphocyte-associated protein 4 (CTLA4), a receptor located on the surface of specific immune cells including T cells, which is responsible for dampening the immune response. When CTLA4 binds to its ligands CD80 (also known as B7-1) and CD86 (also known as B7-2) on antigen-presenting cells (APCs), it inhibits the activation and proliferation of T cells. This is accomplished by competition with CD28, a co-stimulatory molecule crucial for T cell activation. In normal states, this intricate process helps prevent undesired autoimmune responses [109]. In cancer, CTLA4 is harnessed by cancer cells to hinder the antitumor immune response through manipulation of the expression of CTLA4 ligands (CD80 and CD86) on APCs within the tumor microenvironment, enabling CTLA4 on regulatory T cells (Tregs) to suppress cytotoxic T cell activity.

Additionally, CTLA4 competes with CD28 for binding to CD80 and CD86 on APCs, inhibiting T cell activation and resulting in immune inactivation. Furthermore, CTLA4 on Tregs enhances their immunosuppressive functions, further diminishing the antitumor immune response [110].

Liu and colleagues examined 1024 glioma patients and found higher CTLA4 expression in aggressive gliomas, particularly those with higher grades, IDH-wild type status, and the mesenchymal molecular subtype. Furthermore, a robust connection emerged between CTLA4 expression and immune cell infiltration within the glioma microenvironment, signifying potential effects on antitumor immune responses. The study found that lower CTLA4 expression in glioma patients was associated with significantly extended overall survival, underscoring its potential as a valuable prognostic marker [111].

Anti-CTLA4 treatment has demonstrated efficacy in multiple murine models of GBM, with significant reductions in tumor burden and survival benefits that outperformed anti-PD-1 treatment [112]. Anti-CTLA4 treatment also increased the presence of CD4+ T helper cells in GBM. There is, therefore, great interest in the application of CTLA4 inhibitors in GBM.

Antibodies targeting human CTLA4, such as ipilimumab (IPI), have shown significant and long-lasting protective effects against melanoma. In fact, ipilimumab was the first treatment to extend the overall survival of patients with advanced melanoma in a randomized clinical setting [113]. Its remarkable therapeutic benefits have led to ongoing clinical trials to explore its potential in treating various other types of cancer. Recently, the combination of nivolumab (NIVO), a PD-1 blocking monoclonal antibody, and ipilimumab, has been approved for treating various cancer types. The CheckMate 067 trial, a phase III study, demonstrated that nivolumab plus ipilimumab or nivolumab alone had significantly better response rates, longer progression-free survival, and longer overall survival compared to ipilimumab alone in advanced melanoma patients [114]. Combination therapy with nivolumab plus ipilimumab was effective in patients with brain metastases, and some patients were able to discontinue therapy without needing additional treatment for melanoma [114].

In a phase I study assessing the safety of IPI and NIVO, either individually or in combination, in patients with newly diagnosed GBM receiving adjuvant temozolomide (TMZ) treatment, researchers found that IPI and NIVO, whether used alone or in combination, were well-tolerated, with a 16% rate of Grade 4 adverse events. Notably, there were no Grade 5 events, and combination therapy did not result in increased toxicity compared to single agents. These encouraging findings have laid the foundation for subsequent efficacy trials testing checkpoint inhibitor combinations in GBM [115]. Duerinck and colleagues recently demonstrated an innovative approach to recurrent GBM through localized injection of a combination of IPI and NIVO directly into the brain tissue following resection, with encouraging long-term overall survival outcomes. This localized approach demonstrated a reduced incidence of immune-related adverse events and good patient tolerance. While most patients with radiological progression had unfavorable outcomes, a select few showed tumor regressions and long-term survival. This approach offers cost-effectiveness by lower doses and lays the groundwork for potential combinations with other immunotherapies to enhance recurrent GBM treatment [116].

### 5.3. Other Immune Checkpoint Inhibitor Targets

**Figure 2:**
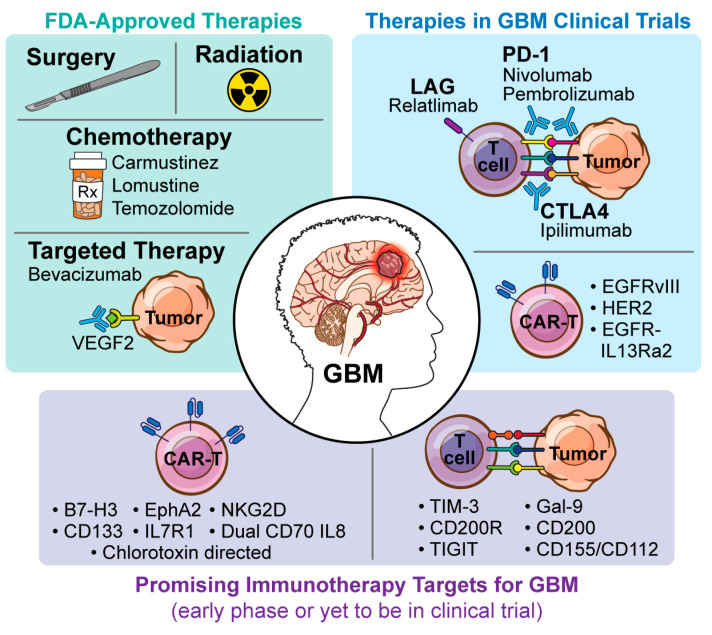
Promising immunotherapy targets for GBM.

Emerging pre-clinical studies are shedding light on several lesser known but potentially promising immune checkpoint blockade targets within the GBM TME (Figure 2). Among these, T cell immunoreceptor with Ig and ITIM domain (TIGIT), lymphocyte activation gene 3 (LAG3), and OX-2 membrane glycoprotein (also known as CD200) are particularly promising therapeutic targets.

TIGIT is mainly expressed on tumor-infiltrating effector T cells, regulatory T cells, and NK cells within various tumor types, and exerts its immunosuppressive effects in T cells by competing with the CD226 receptor for binding to the shared ligands CD155 and CD112 [117,118,119,120,121,122]. Moreover, these ligands are predominantly expressed by tumor cells, tumor-infiltrating myeloid cells, and antigen-presenting cells (APCs), underlining TIGIT’s role in TME-specific T cell immunosuppression [121]. In the context of GBM, studies have noted that higher TIGIT expression is correlated with poor patient progression-free and overall survival [123,124,125]. Moreover, preclinical studies on dual PD-1 and TIGIT immunotherapy have shown improved survival in murine GBM models [124].

LAG3 is a regulatory receptor mostly expressed by activated effector T cells, regulatory T cells, NK cells, and B cells [126,127,128]. LAG3 mediates the suppression of T cells upon binding with Fibrinogen-like protein 1 (FGL1), which regulates T cell activation, proliferation, and cytokine production [128,129]. In a global phase I/II clinical trial involving LAG3 in untreated advanced melanoma, patients treated with anti-LAG3 in combination with anti-PD-1 exhibited a median progression-free survival of 10.1 months compared to 4.6 months in an anti-PD1-only treatment group [130]. Of note, numerous studies have shown that tumor-infiltrating lymphocytes in GBM express LAG3 [131,132,133]. Clinical trials evaluating the therapeutic potential of anti-LAG3 alone, and anti-CD137 alone or in combination with anti-PD1, are currently ongoing in patients with recurrent GBM [134]. Additionally, in murine models, combination treatment with anti-LAG3 and anti-PD1 showed a trend toward improved survival compared to anti-LAG3 alone, and LAG3 knockout mice showed an improved response to treatment with anti-PD-1 compared to their wild-type counterparts [132]. Overall, the presence of LAG3-expressing TILs in GBM, along with the clinical benefits of LAG3 blockade in extracranial tumors and GBM murine models, emphasizes LAG3 as a promising novel target for GBM patients.

Finally, the CD200/CD200R axis represents a critical immunoregulatory pathway for the maintenance of immune tolerance within healthy tissue [135]. CD200R is abundantly expressed by monocytes, myeloid cells, neutrophils, and subsets of T cells [136,137]. Its ligand, CD200, is expressed by T cells, B cells, macrophages, microglia, endothelial cells, neurons, stromal cells, and tumor cells [138]. In the normal brain, CD200 is expressed by neurons to modulate macrophage and microglia activation, and loss of CD200 expression in murine models has been shown to result in the development of autoimmune encephalomyelitis (EAE) [139]. CD200 expression has been reported in a variety of solid and liquid tumors, including GBM [135,140]. Studies in multiple myeloma show that tumor cells co-cultured with primary T cells are more likely to survive when expressing CD200, which is also associated with increased Treg levels and reduction in T cell effector function [141]. The therapeutic impact of targeting CD200 in GBM is currently being investigated in a phase I clinical trial using CD200AR-L in combination with imiquimod and the GBM6-AD vaccine. Preliminary results comparing pre- and post-vaccination show increased intratumoral CD4 and CD8 T cells, and downregulation of PD-1/PD-L1 and CTLA4 expression on effector T cells and myeloid cells [140]. While targeting of CD200, TIGIT, and LAG3 is less established in GBM treatment, these targets should be highly considered for future drug development as they appear to be present in GBM and may serve as a strong alternative target should a patient’s tumor express low amounts of PD-1/PD-L1 or CTLA4.

## 6. Discussion

Immunotherapy has demonstrated significant therapeutic potential in multiple cancers but has been largely unsuccessful in the treatment of GBM. Our review highlights specific barriers to immunotherapy in GBM, either physical or immunological, and discusses how these factors enable the growth and proliferation of GBM. Other reviews have highlighted the immunosuppressive components of the GBM microenvironment; however, there is a paucity of literature that highlights potential therapies that may combat these components [142,143].

The unique and challenging nature of GBM necessitates a paradigm shift in both our understanding of this malignancy and the mechanisms GBM utilizes to evade treatment. The current limitations, stemming from a lack of modification and development in GBM therapeutics, coupled with an incomplete understanding of the genetic intricacies of GBM tumors and their capacity to induce CNS-specific immunosuppression, underscore the urgent need for innovative strategies.

While neoadjuvant treatments have demonstrated success in restoring T cell response in non-CNS tumors, their limitations in CNS applications emphasize the necessity for the development of GBM-specific therapeutics [11,144,145]. The current golden standard-of-care measures, such as surgery and radiation therapy, remain noncurative, with limited efficacy. The most targeted therapy to date, TMZ, has exhibited only marginal effectiveness in a subset of GBM patients over the past two decades [11,144,145]. Concurrently, supportive treatments such as dexamethasone come with significant drawbacks, as this therapy impairs T cell responses. On the other hand, controversies surrounding the benefits and implications of radiotherapy, including its potential role in inducing T cell exhaustion and tumor progression, emphasize the need for strategic utilization of radiotherapy for GBM treatment [11,144,145].

Clinical trials have had poor results in adult GBM patients. Often, many of these trials are evaluating novel immune-based therapies that are aimed at inducing a robust, T cell-driven, antitumor immune response. However, it has been postulated that many patients enrolled in these trials may not experience a strong response to immune-based therapies as these patients may be immunosuppressed by chemoradiation, corticosteroids, and multiple surgeries. A new clinical trial (NCT04817254) seeks to evaluate whether it is possible to determine which patients are more likely to respond to immune checkpoint inhibition. Specifically, the research team is acquiring peripheral blood samples from GBM patients prior to initiating treatment with dual immune checkpoint inhibitor therapy. T cells isolated from these pre-treatment blood samples are incubated with Tosylactivated beads, both with and without ipilimumab measuring proliferation as the metric of activation. Longitudinal blood samples are also evaluated for evidence of response to the clinical treatment to then determine if development of a peripheral blood immune response is associated with improved survival. The response to the bead assay will be compared with the clinical response to checkpoint treatment to determine if the assay is a good predictor of patient response to this immunotherapy. If successful, this study would support that immune checkpoint treatment may benefit a subgroup of patients with GBM and that there may be an assay to enrich for this subgroup of patients [146]. Future randomized clinical trials to confirm these findings would then be able to use the bead assay to enrich for patients capable of mounting a peripheral blood response to the immunotherapy, thereby potentially increasing the effect size and reducing the accrual requirements for the clinical trial.

Despite the common utilization of genetic profiling of non-CNS tumors, GBM-specific genetic characteristics remain significantly understudied. Profiling the GBM genome holds immense potential for defining key tumor features, including identifying tumor-specific metabolic pathways, metastatic and progression markers, and immunosuppressive traits. Furthermore, temporal and spatial profiling promises to unveil not only tumor-specific pathways, but also the activation of different pathways over time and in distinct anatomical locations [147]. Recently, single cell RNA sequencing and bulk RNA sequencing have been utilized to study human GBMs, allowing four cellular states to be observed in these tumors: astrocyte-like (AC-like), oligodendrocyte precursor cell-like (OPC-like), neural progenitor cell-like (NPC-like), and mesenchymal-like (MES-like) [5]. The findings of Tirosh and colleagues have shown that MES-like programs in GBM are associated with high proportions of macrophages, microglia, and cytotoxic T cells, while AC-like, OPC-like, and NPC-like programs were associated more closely with neuronal/glial lineage progenitor cells [148,149]. In future studies, it will be crucial to explore the understanding of how these different cell states that exist in GBM impact the TME and patient response to treatment.

The unique challenge of immunosuppression in GBM, and how this may be variable across tumor subtypes, compounded by the tumor’s localization in the CNS, demands a holistic understanding of the interplay between the tumor, the immune system, and the CNS microenvironment [146]. T cell-specific therapies currently exhibit low therapeutic efficacy due to continuous tumor escape mechanisms. A promising avenue for overcoming these challenges may involve combining therapies to restore T cell responses while concurrently targeting epigenetic-mediated immune suppression or tumor metabolism in GBM.

## 7. Conclusions

In conclusion, advancing GBM research and treatment necessitates a multifaceted approach that incorporates GBM-specific therapeutics, genetic profiling, determining patient likelihood for treatment response, and a careful understanding of the tumor microenvironment. Only through such comprehensive efforts can we hope to uncover novel insights, identify effective therapeutic targets, restore CNS T cell antitumor response, and ultimately improve outcomes for GBM patients.

## Figures and Tables

**Table 1:** Clinical trials evaluating CAR-T cell therapy in gliomas.

NCT Number	Other IDs	Cancer Type	Study Results	CAR-T	Phases	Study Status	Treatment
NCT02664363	Pro00069444	Newly Diagnosed GBM During Lymphopenia	Y	EGFRvIII CAR-T cells	PHASE 1	T	EGFRvIII CAR-T cells
NCT01454596	110266|11-C-0266	Malignant Gliomas Expressing EGFRvIII	Y	EGFRvIII CAR-T cells	PHASE 1|PHASE 2	C	1/Phase I Arm: Escalating doses of EGFRvIII CAR-T cells transduced peripheral blood lymphocytes (PBL) 2/Phase II Arm: Maximum tolerated dose of anti-EGFRvIII CAR-T transduced PBL established in Phase 1
NCT06482905	TX103T-RG008	Recurrent or Progressive Grade 4 Glioma	N	B7-H3 CAR-T (TX103)	PHASE 1	NR	Cohort A: Single delivery routes: Anti-B7-H3/TX103 CAR-T cells Cohort B: Dual delivery route: Anti-B7-H3/TX103 CAR-T cells
NCT06186401	23704|5U19CA264338-03	Newly diagnosed EGFRvIII+ Glioblastoma	N	EGFRvIII synNotch Receptor Induced Anti-EphA2/IL-13R alpha2 CAR (E-SYNC) T Cells	PHASE 1	R	Cohort 1 Starting Dose: E-SYNC CAR-T cells Cohort 1 Dose-escalation: E-SYNC CAR-T cells Cohort 2 Tissue analysis cohort: EGFRvIII H-score of >=250: Maximum tolerated E-SYNC CAR-T cells dose EGFRvIII H-score of <250: Recommended E-SYNC CAR-T cells dose based on results from cohort 1
NCT05868083	SNC-109-101	Recurrent Glioblastoma	N	SNC-109 CAR-T cells	PHASE 1	R	SNC-109 CAR-T cell therapy
NCT05802693	A03728	Recurrent Glioblastoma	N	EGFRvIII CAR-T cells	EARLY PHASE 1	NR	EGFRvIII CAR-T cell therapy
NCT05660369	22-175	Glioblastoma	N	CARv3-TEAM-E T Cells	PHASE 1	R	Safety Run-In Phase: 1 infusion of CARv3-TEAM-E Arm 1 Recurrent GBM, EGFRvIII Positive: CARv3-TEAM-E Arm 2 Newly Diagnosed GBM, EGFRvIII Positive: CARv3-TEAM-E Arm 2 Newly Diagnosed GBM, EGFRvIII Negative: CARv3-TEAM-E
NCT05627323	CHM-1101-001	MMP2+ Recurrent or Progressive Glioblastoma	N	CHM-1101 CAR-T cells	PHASE 1	NR	Arm 1: Dose level 1 CHM-1101 CAR-T cells Arm 2: Dose level 2 CHM-1101 CAR-T cells
NCT05577091	TTSW2021-01	Recurrent Glioblastoma	N	Autologous Tris-CAR-T cells	PHASE 1	R	Autologous Tris-CAR-T cells
NCT05474378	IRB-65002|NCI-2022-06043	Recurrent Glioblastoma	N	B7-H3 CAR-T cells	PHASE 1	R	Arm 1 Dose escalation: B7-H3CAR-T Arm 2 Dose Expansion: B7-H3CAR-T
NCT05366179	LCCC2059-ATL	Recurrent or Refractory Glioblastoma	N	B7-H3 CAR-T cells	PHASE 1	R	CAR.B7-H3T cells therapy
NCT05353530	IRB202200057	CD70+ Adult GBM	N	8R-70CAR-T cells	PHASE 1	R	Single dose of 8R-70CAR-T cell therapy
NCT05241392	TX103T-IG005	Recurrent Glioblastomas	N	B7-H3-targeting CAR-T cells	PHASE 1	R	B7-H3-targeting CAR-T cell therapy
NCT05131763	Fudan-Changchun	Relapsed/Refractory NKG2DL+ Solid Tumors	N	NKG2D-based CAR-T cells	PHASE 1	U	NKG2D-based CAR-T cell therapy
NCT05063682	6678EGFRvIII	Leptomeningeal Glioblastoma	N	EGFRvIII-CAR-T cells	PHASE 1	U	EGFRvIII-CAR-T cell therapy
NCT04661384	19497|NCI-2020-06010|19497|P30CA033572	Adult with Leptomeningeal Glioblastoma, Ependymoma or Medulloblastoma	N	IL13Ralpha2-CAR-T cells	PHASE 1	R	IL13Ralpha2-CAR-T cells therapy
NCT04385173	SAHZJU-BP102	Recurrent and Refractory Glioblastoma	N	B7-H3 CAR-T	PHASE 1	R	B7-H3 CAR-T between cycles of Temozolomide treatment
NCT04270461	JiujiangUH	Relapsed/Refractory NKG2DL+ Solid Tumors	N	NKG2D-based CAR-T cells	PHASE 1	W	NKG2D-based CAR-T cells therapy
NCT04214392	19309|NCI-2019-08393|19309	MMP2+ Recurrent or Progressive Glioblastoma	N	Chlorotoxin (EQ)-CD28-CD3zeta-CD19t-expressing CAR-T-lymphocytes	PHASE 1	R	Arm 1: Chlorotoxin-CD28-CD3z-CD19t-expressing CAR-T cells, ICT delivery Arm 2: Chlorotoxin-CD28-CD3z-CD19t-expressing CAR-T cells, ICT/ICV dual delivery
NCT04077866	SAHZJU-RCT-BP102	Recurrent or Refractory Glioblastoma	N	B7-H3 CAR-T	PHASE 1|PHASE 2	R	Arm 1: Temozolomide alone Arm 2: Temozolomide + B7-H3 CAR-T therapy
NCT04045847	Chen Zhinan-2	Recurrent Glioblastoma	N	CD147-CART	EARLY PHASE 1	U	CD147-CAR-T therapy
NCT04003649	18251|NCI-2018-02764|18251|R01CA236500	Resectable Recurrent Glioblastoma	N	IL13Ralpha2 CAR-T cells	PHASE 1	R	Arm I: Nivolumab + Ipilimumab + IL13Ralpha2 CAR-T cells Arm II: Nivolumab + IL13Ra2 CAR-T cells Arm III: IL13Ra2 CAR-T cells
NCT03726515	831706, UPCC 13318	Newly Diagnosed, MGMT-Unmethylated Glioblastoma	N	CART-EGFRvIII T cells	PHASE 1	C	CART-EGFRvIII + Pembrolizumab
NCT03283631	Pro00083828|5P50CA190991-03	Recurrent Glioblastoma	N	EGFRvIII-CARs	PHASE 1	T	EGFRvIII-CARs therapy
NCT03170141	GIMI-IRB-17003	Glioblastoma	N	Antigen-specific IgT cells	PHASE 1	R	Antigen-specific IgT cells therapy
NCT02937844	SBNK-2016-016-01	Recurrent Glioblastoma	N	Anti-PD-L1 CSR T cells	PHASE 1	U	Anti-PD-L1 CAR-T cells therapy
NCT02844062	SBNK-2016-015-01	Recurrent Glioblastoma	N	anti-EGFRvIII CAR-T cells	PHASE 1	U	Anti-EGFRvIII CAR-T cells therapy
NCT02209376	UPCC 35313, 820381	EGFRVIII+ Glioblastoma	N	CART-EGFRvIII T cells	PHASE 1	T	CART-EGFRvIII T cells therapy

T = terminated; C = completed; NR = not recruiting; R = recruiting; W = withdrawn; U = unknown; Y = yes; N = no.

**Table 2:** Clinical trials evaluating immune checkpoint blockade in gliomas.

NCT Number	Other IDs	Cancer Type	Study Results	Phases	Study Status	Treatment
NCT05235737	PIRG	Newly Diagnosed Glioblastoma	N	PHASE 4	R	Arm 1: Pembrolizumab as a neoadjuvant and adjuvant therapy to standard chemo-radiotherapy Arm 2: Pembrolizumab as a neoadjuvant therapy to standard chemo-radiotherapy Arm 3: Standard chemo-radiotherapy
NCT02617589	CheckMate 498	Newly Diagnosed Adult Subjects with Unmethylated MGMT GBM	Y	PHASE 3	C	Arm 1: Nivolumab + Radiation Arm 2: Temozolomide + Radiation
NCT02017717	CheckMate 143	Recurrent Glioblastoma	Y	PHASE 3	C	Arm 1: Nivolumab Arm 2: Bevacizumab
NCT02667587	CheckMate548	Newly Diagnosed Adult Subjects with MGMT-Methylated GBM	Y	PHASE 3	C	Arm 1: Nivolumab + Radiation + Temozolomide Arm 2: Placebo + Radiation + Temozolomide
NCT06556563	EF-41	Newly Diagnosed Glioblastoma	N	PHASE 3	NR	Arm 1: Optune® device + Temozolomide + Pembrolizumab Arm 2: Optune® device + Temozolomide + Placebo
NCT04396860		Newly Diagnosed Adult Subjects with MGMT-Methylated GBM	Y	PHASE 2|PHASE 3	NR	Arm 1: Radiation therapy + Temozolomide Arm 2: Radiation therapy + Ipilimumab + Nivolumab
NCT03430791		Recurrent Glioblastoma	Y	PHASE 2	T	Arm 1: Nivolumab monotherapy Arm 2: Nivolumab + Ipilimumab
NCT02794883		Recurrent Malignant Glioma	Y	PHASE 2	C	Arm 1: Tremelimumab Only Arm 2: MEDI4736 Only Arm 3: Tremelimumab + MEDI4736
NCT03018288		Newly diagnosed GBM without MGMT-Methylation	Y	PHASE 2	T	Arm 1: Vaccine Arm 2: Placebo Arm 3: Ancillary Treatment
NCT02337686		Recurrent Malignant Glioma	Y	PHASE 2	NR	Pembrolizumab + Surgery
NCT03661723		Bevacizumab Naïve and Bevacizumab Resistant Recurrent Glioblastoma	Y	PHASE 2	NR	Arm 1: COH A - Dose Level 0 (200 mg Pembrolizumab once every 3 Weeks + 2 Weeks of Radiation) Arm 2: COH B - Dose Level 0 (200 mg Pembrolizumab + 15 mg/kg Bevacizumab once every 3 Weeks + 2 Weeks of Radiation)
NCT02337491		Recurrent Malignant Glioma	Y	PHASE 2	C	Arm 1: Pembrolizumab + Bevacizumab Arm 2: Pembrolizumab
NCT03367715		Newly Diagnosed, Unmethylated MGMT Glioblastoma	Y	PHASE 2	C	Nivolumab + Ipilimumab + Short-course radiation therapy
NCT04013672		Glioblastoma at First Recurrence	Y	PHASE 2	C	Arm 1: Have not received immunotherapy Arm 2: Have failed prior anti-PD-1 therapy
NCT05879120		Recurrent Glioblastoma	N	PHASE 2	NR	Arm 1: Neoadjuvant Pembrolizumab Arm 2: Exablate MRgFUS + neoadjuvant Pembrolizumab
NCT05463848		Recurrent Glioblastoma	N	PHASE 2	R	Cohort 1 (Safety Lead In): Pembrolizumab plus Olaparib and Temozolomide Cohort 2 (Surgical Cohort): Arm A - Pembrolizumab plus Olaparib and Temozolomide Cohort 3 (Surgical Cohort): Arm B - Pembrolizumab monotherapy
NCT05909618	14	Glioblastoma and Melanoma with Brain Metastases	N	PHASE 2	R	Cohort 1: Crizanlizumab + Nivolumab in Metastatic melanoma with brain metastases who failed immunotherapy Cohort 2: Crizanlizumab + Nivolumab in Patients with recurrent or progressing Glioblastoma following radiation and Temozolomide Cohort 3: Crizanlizumab + Nivolumab in Patients with newly diagnosed Glioblastoma
NCT03014804		Recurrent Glioblastoma	N	PHASE 2	W	Arm 1: DCVax-L Arm 2: DCVax-L + Nivolumab
NCT04225039		Recurrent Glioblastoma	Y	PHASE 2	NR	Arm 1: GITR + INCMGA00012 (anti-PD-1) + SRS Arm 2: GITR + INCMGA00012 (anti-PD-1) + SRS + Surgery Arm 3: GITR + INCMGA00012 (anti-PD-1) + Surgery
NCT06328036		Recurrent Glioblastoma	N	PHASE 2	NR	Arm 1: Neoadjuvant Atezolizumab + Tiragolumab Arm 2: Neoadjuvant Tiragolumab Arm 3: Neoadjuvant Atezolizumab Arm 4: No neoadjuvant drug
NCT04817254		Newly Diagnosed Glioblastoma or Gliosarcoma	N	PHASE 2	R	Arm 1: Nivolumab + Ipilimumab 1mg/kg + Temozolomide Arm 2: Nivolumab + Ipilimumab 3 mg/kg + Temozolomide
NCT03452579		Recurrent Glioblastoma	N	PHASE 2	NR	Arm 1: Nivolumab + Standard dose Bevacizumab 10 mg/kg Arm 2: Nivolumab + Low dose Bevacizumab 3 mg/kg
NCT06325683		Recurrent Glioblastoma	N	PHASE 2	NR	Arm 1: Nivolumab + Relatlimab Arm 2: Lomustine
NCT06558214	OPTIMUS PRIME	Recurrent Glioblastoma	N	PHASE 2	NR	Arm 1: Optune GIO^®^ pre-MLA; MLA; followed by Optune GIO^®^ + Pembrolizumab post MLA Arm 2: Optune GIO^®^ + Pembrolizumab pre-MLA; MLA; followed by Optune GIO^®^ + Pembrolizumab post MLA
NCT04118036		Recurrent Glioblastoma	N	PHASE 2	W	Arm 1: Pembrolizumab + Abemaciclib + Surgery Arm 2: Pembrolizumab + Abemaciclib + non-surgery
NCT03797326		Previously Treated Subjects with Selected Solid Tumors (LEAP-005)	N	PHASE 2	NR	Arm 1: Pembrolizumab + Lenvatinib Arm 2: Lenvatinib monotherapy
NCT03197506		Newly Diagnosed Glioblastoma	N	PHASE 2	S	Arm 1: Pembrolizumab + Surgery + Temozolomide + Radiation Arm 2: Pembrolizumab + Temozolomide + Radiation therapy
NCT04195139	NUTMEG	Newly Diagnosed Elderly Patients with Glioblastoma (NUTMEG)	N	PHASE 2	NR	Arm 1: Radiotherapy + Nivolumab and Temozolomide Arm 2: Radiotherapy + Temozolomide
NCT03743662		Recurrent MGMT Methylated Glioblastoma	N	PHASE 2	NR	Arm 1: Re-irradiation + Bevacizumab + Nivolumab + Recurrent Glioblastoma + No Surgery Arm 2: Re-irradiation + Bevacizumab + Nivolumab + Recurrent Glioblastoma + Surgery
NCT04729959		Recurrent Glioblastoma	N	PHASE 2	R	Arm 1: Tocilizumab + Atezolizumab + FSRadiation Arm 2: Tocilizumab + Atezolizumab + FSRadiation + surgery Arm 3: Tocilizumab + Atezolizumab + FSRadiation + surgery
NCT03890952		Recurrent Glioblastoma	N	PHASE 2	NR	Arm B: Nivolumab and Bevacizumab in patients not undergoing salvage surgery Arm A: Nivolumab and Bevacizumab in patients undergoing salvage surgery
NCT02798406	CAPTIVE	Recurrent Glioblastoma or Gliosarcoma (CAPTIVE/KEYNOTE-192)	N	PHASE 2	C	DNX-2401 + Pembrolizumab
NCT05465954		Recurrent Glioblastoma	N	PHASE 2	R	Efineptakin alfa + Pembrolizumab before and after surgery
NCT03347617		Glioblastoma	N	PHASE 2	NR	Ferumoxytol MRI + Pembrolizumab
NCT05074992	NeAT Glio	Newly Diagnosed Glioblastoma	N	PHASE 2	T	Ipilimumab
NCT04479241		Recurrent Glioblastoma	N	PHASE 2	NR	Lerapolturev + Pembrolizumab
NCT02550249	Neo-Nivolumab	Glioblastoma	N	PHASE 2	C	Nivolumab
NCT04145115		Somatically Hypermutated Recurrent WHO Grade 4 Glioma	N	PHASE 2	S	Nivolumab + Ipilimumab
NCT03718767		IDH-Mutant Gliomas with and without Hypermutator Phenotype	N	PHASE 2	R	Nivolumab in IDH-mutant gliomas patients with and without HMP in response
NCT03899857	PERGOLA	Newly Diagnosed Glioblastoma	N	PHASE 2	NR	Pembrolizumab + Temozolomide-based chemoradiation
NCT06069726	MOAB	Recurrent Glioblastoma	N	PHASE 2	R	Pre-Surgery Atezolizumab
NCT03405792	2-THE-TOP	Newly Diagnosed Glioblastoma	Y	PHASE 2	NR	Arm 1: Optune system combined with Temozolomide + Pembrolizumab Arm 2: Historical control

T = terminated; C = completed; NR = not recruiting; R = recruiting; W = withdrawn; S = suspended; Y = yes; N = no.

## Data Availability

All articles reviewed in this manuscript have been cited in the references.
